# Positive Body Image and Sexual Functioning in Dutch Female University Students: The Role of Adult Romantic Attachment

**DOI:** 10.1007/s10508-015-0511-7

**Published:** 2015-03-17

**Authors:** Femke van den Brink, Monique A. M. Smeets, David J. Hessen, Liesbeth Woertman

**Affiliations:** 1 Division of Clinical and Health Psychology, Department of Behavioral Sciences, Utrecht University, Heidelberglaan 1, 3508 TC Utrecht, The Netherlands; 2 Division of Methodology and Statistics, Department of Social Sciences, Utrecht University, Utrecht, The Netherlands

**Keywords:** Body image, Body appreciation, Sexual functioning, Romantic attachment

## Abstract

This study focused on links between romantic attachment, positive body image, and sexual functioning. Dutch female university students (*N* = 399) completed an online survey that included self-report items about body appreciation, sexual functioning, and romantic attachment. A proposed conceptual model was tested using structural equation modeling and a good fit to the data was found. Results revealed that attachment avoidance in a romantic context was negatively related to sexual arousal, vaginal lubrication, the ability to reach orgasm, and sexual satisfaction. Attachment anxiety was negatively related to body appreciation which, in turn, was positively related to sexual desire and arousal. Findings indicated that romantic attachment is meaningfully linked to body appreciation and sexual functioning. Therefore, the concept of adult attachment may be a useful tool for the treatment of sexual problems of young women.

## Introduction

Body image is best characterized as a multidimensional construct encompassing self-perceptions, attitudes, feelings, and behaviors about one’s physical appearance (Cash, 2002).

The main focus in body image research has been on negative aspects of this construct (e.g., body dissatisfaction). Negative body image has been found to be associated with several aspects of poor mental health, such as disordered eating behaviors (Polivy & Herman, 2002) and depression (Stice, Hayward, Cameron, Killen, & Taylor, 2000). From these findings, it could be inferred that positive body image, which refers to the love, respect, acceptance, and appreciation held for one’s body (Tylka, 2011), is associated with healthy psychological functioning. However, characteristics, predictors, and outcomes of positive body image may not be simply the opposite of those of negative body image (e.g., healthy eating behaviors instead of disordered eating behaviors). Moreover, positive body image is not synonymous with the absence of negative body image (e.g., Avalos, Tylka, & Wood-Barcalow, 2005). Positive body image is reflected by the absence of negative body attitudes and dissatisfaction, and the presence of favorable opinions of and positive feelings about the body.

Consistent with the positive psychology movement, it is important to not just the study of absence of pathology, weakness, and damage, but to acknowledge the role of positive traits that contribute to and maintain overall psychological health (Seligman & Csikszentmihalyi, 2000). Therefore, the focus in this study was on positive body image. Unless we fully understand the determinants of positive body image, clinical interventions cannot be expected to be completely successful in reducing negative body image and building positive body image.

Previous studies have found that positive body image was associated with several features of better overall adjustment and mental health, such as higher levels of self-esteem, more criticism on unrealistic beauty ideals, and more negative attitudes toward cosmetic surgery (Avalos et al., 2005; Grogan, 2010; Holmqvist & Frisén, 2012; Swami, 2009; Wood-Barcalow Tylka, & Augustus-Horvath, 2010). An equally important element of overall health and happiness is healthy and satisfying sexual functioning, as sexuality plays a considerable role in intimate relationships and is an important aspect of emotional and psychological health (Bridges, Lease, & Ellison, 2004; Levin, 2007).

Relationships between body image and female sexual functioning have been investigated mostly in negative terms. Although it is clear that body image issues can negatively affect different domains of sexual functioning (for a review, see Woertman & van den Brink, 2012), little is known about associations between sexual functioning and positive aspects of body image. To our knowledge, only two studies focused specifically on aspects of positive body image and relationships with female sexuality. Satinsky, Reece, Dennis, Sanders, and Bardzell (2012) found in a sample of North American adult women that body appreciation positively predicted sexual arousal, the ability to reach orgasm, and sexual satisfaction aspects of sexual functioning. Likewise, van den Brink, Smeets, Hessen, Talens, and Woertman (2013) found that Dutch female university students who reported overall body satisfaction reported better general sexual functioning than women who were neutral about their bodies. These results indicate that positive body image, above and beyond the absence of negative body image, is important for positive sexual experiences.

There might be additional factors that impact associations between positive body image and sexual functioning. Identifying such factors is important in selecting the appropriate targets for treatment intervention in the context of sexual dysfunction and of improving women’s sexual health and well-being. To this end, we examined links of attachment in romantic relationships with positive body image and sexual functioning.

Attachment refers to the ways individuals organize their connections to important others (Bowlby, 1969, 1973). It was originally considered as being categorical (i.e., secure, preoccupied, fearful, and dismissing) (Bartholomew & Horowitz, 1991), but is currently often conceptualized as two continuous dimensions that underlie attachment orientations: anxiety (i.e., anxiety and vigilance concerning rejection and abandonment) and avoidance (i.e., discomfort with closeness and dependency or a reluctance to be intimate with others), with low levels of both dimensions suggesting secure attachment (Brennan, Clark, & Shaver, 1998; Fraley & Shaver, 2000). Both anxious and avoidant attachment are associated with difficulties in forming or maintaining healthy relationships with others (Bowlby, 1973).

Body image is strongly influenced by interactions with important others, with romantic partners contributing most strongly in adulthood (Tantleff-Dunn & Gokee, 2002). For example, negative comments and influences by romantic partners contribute to negative body image, whereas supportive communication helps to reduce body image stress and increase self-esteem (Weller & Dziegielewski, 2005). Insecurely attached individuals are likely to make more pessimistic attributions for their partner’s behavior, whenever it activates one’s fears about being rejected or doubts about the trustworthiness of others (Collins, Ford, Guichard, & Allerd, 2006). Therefore, insecure attachment to romantic partners might be associated with more vulnerability for interpreting (ambiguous) body-related comments of the partner in a negative way, resulting in negative feelings towards one’s own body.

Studies that focused on the link between adult attachment and body image primarily examined relationships between aspects of body image and general adult attachment (i.e., attachment experiences in close relationships in general, without specifying whether those close others were romantic partners, parents, friends, etc.) (e.g., Elgin & Pritchard, 2006; Iannantuono & Tylka, 2012). Only a small number of studies focused on body image and attachment in romantic relationships specifically. One study that used a four-category model of adult attachment (i.e., secure, preoccupied, fearful, and dismissing) (Bartholomew & Horowitz, 1991) found that a secure romantic attachment style in adulthood was positively related to a favorable body image, whereas a preoccupied romantic attachment style-characterized by high attachment anxiety but low avoidance-was associated with more body dissatisfaction and dysphoria. In the same study, the two underlying continuous dimensions of romantic attachment (i.e., anxiety and avoidance) were assessed. Anxious romantic attachment was a predictor of body dissatisfaction in a sample of North American female college students, whereas avoidant romantic attachment was not (Cash, Thériault, & Annis, 2004). The results were broadly in line with findings of other studies. Only romantic attachment anxiety was found to be associated with concerns and dissatisfaction about body shape in a samples of female college students (Hardit & Hannum, 2012; Koskina & Giovazolias, 2010). Evans and Wertheim (1998) found in their sample of young adult females that anxious romantic attachment was associated with drive for thinness and general body dissatisfaction. Taken together, previous research suggests that romantic attachment anxiety is associated with multiple aspects of negative body image, but that romantic attachment avoidance is unrelated to negative body image. To our knowledge, no studies have yet examined associations between positive body image and romantic attachment.

In adulthood, romantic partners typically function simultaneously as sexual partners and attachment figures (Hazan, Zeifman, & Middleton, 1994). Empirical studies have supported relationships between adult attachment in romantic relationships and various aspects of sexuality (for a review, see Stefanou & McCabe, 2012). Previous studies in clinical samples showed that both attachment anxiety and avoidance were related to painful experiences during sexual intercourse (Granot, Zisman-Ilani, Ram, Goldstick, & Yovell, 2010) and sexual dissatisfaction (Brassard, Péloquin, Dupuy, Wright, & Shaver, 2012). Studies in community samples found that both attachment anxiety and avoidance were associated with less sexual arousal (Birnbaum, 2007), problems with lubrication (Brassard, Shaver, & Lussier, 2007), lower levels of orgasmic frequency (Cohen & Belsky, 2008), and sexual dissatisfaction (Davis et al., 2006). In samples of female college students, attachment anxiety and avoidance were linked with impaired vaginal orgasm (Costa & Brody, 2011), sexual distress (Stephenson & Meston, 2010a), and negative affect about sexual experiences (Gentzler & Kerns, 2004). In addition, attachment anxiety was found to be related to less sexual satisfaction in female undergraduates (Stephenson & Meston, 2011).

The current study investigated associations of romantic attachment with positive body image and sexual functioning in young female university students. In early adulthood, romantic partners typically start to serve as important attachment figures (Fraley & Shaver, 2000). During this time, dating relationships are generally transformed into more serious romantic relationships and the attachment and caregiving features of romantic relationships become salient (Furman, 2002). Sexual activity often takes place within the context of these relationships (Willetts, Sprecher, & Beck, 2004). The increasing impact of a romantic partner in early adulthood makes this period in life of particular interest.

Based on the previous studies in this field-for most part of samples of college women-we posited that romantic attachment has important links with positive body image and female sexual functioning. We expected positive body image to be related to sexual functioning (van den Brink et al., 2013) (Hypothesis 1). Furthermore, romantic attachment-related anxiety was expected to be linked with positive body image (e.g., Cash et al., 2004) (Hypothesis 2). Additionally, we expected romantic attachment anxiety and avoidance to be related to lower sexual functioning scores (e.g., Birnbaum, 2007) (Hypothesis 3 and 4, respectively). Furthermore, previous research found that the anxiety and avoidance dimension of romantic attachment were interrelated (Fraley, Heffernan, Vicary, & Brumbaugh, 2011). This indicates that individuals who are highly anxious in relationship with a romantic partner also tend to avoid intimacy with this person and vice versa. We therefore predicted that romantic attachment anxiety and avoidance would be associated (Hypothesis 5).

## Method

### Participants

The participants were recruited via the Internet. The Website of Utrecht University provided a link to the questionnaire. The program “Net questionnaires” was used to create the online questionnaire. Students signed up for participation via a special website only accessible to students listing all available studies. Criteria for participation were female gender, university student, between 18 and 35 years old, and sexually active (with a partner, now or in the past). When opening the link, participants first completed an informed consent form. The questionnaires measured body appreciation, attachment anxiety and avoidance in romantic relationships, and sexual functioning. Demographic and personal questions were also included. These items asked participants’ age, height, weight, religious affiliation, and sexual orientation. Participants were also asked if they were currently involved in a romantic relationship with a partner and if they were sexually active with their partner. Participants received course credit for participating in the study. On average, it took 25 min to complete the questionnaire.

A total of 399 Dutch female university students participated in this study. Age ranged from 18 to 29 years (*M* = 21.70, *SD* = 1.98). The large majority of the sample (72.8 %, *n* = 291) reported no religious affiliation, 23.3 % (*n* = 93) were of Christian religion, and 4.1 % (*n* = 16) were otherwise religious. In the total sample, 94.8 % (*n* = 379) were heterosexual, 1.3 % were homosexual (*n* = 5), and 4.0 % (*n* = 16) were bisexual. Most participants had a current romantic partner and were sexually active with their partner (66.7 %, *n* = 266). Participants’ Body Mass Index (BMI) was calculated from self-reported weight and height (kg/m^2^). Percentages of underweight (BMI < 18.5 kg/m^2^), normal weight (BMI 18.5–25 kg/m^2^), overweight (BMI 25–30 kg/m^2^), and obese (BMI > 30 kg/m^2^) participants were also calculated. The majority of the participants were in the normal weight range (85.7 %, *n* = 342), 4.0 % (*n* = 16) were underweight, 8.3 % (*n* = 33) were overweight, and 2.0 % (*n* = 8) were obese.

Comparisons with national census data showed that the sample was overrepresented by non-religious participants. In the Netherlands, 55 % of young women (aged 18–25 years) with higher education reported being nonreligious, whereas 31 % reported a Christian religion (Centraal Bureau voor de Statistiek, 2009). Moreover, our sample was underrepresented by overweight participants. In the general population of young Dutch women (aged 18–25 years), 27.4 % were found to be overweight and 66.8 % were in the normal weight range (Centraal Bureau voor de Statistiek, 2011).

### Measures

All scales were translated from English to Dutch with the translate-retranslate method (retranslation by native speaker), unless otherwise stated.

#### Positive Body Image

Positive body image was measured was assessed by measuring body appreciation using the Dutch version of the Body Appreciation Scale (BAS) (Avalos et al., 2005). The scale consists of 13 5-point *never*-*always* Likert items. One example of an item is: “I respect my body.” Scores were averaged to obtain an overall body appreciation score. Higher scores indicated greater body appreciation. This scale has been reported to be internally reliable in a sample of young females (*α* = .94; Avalos et al., 2005). In the present study, Cronbach’s alpha for this scale was .88. Means and *SD*s for the current sample are shown in Table 1.

**Table 1:** Means and SDs for the positive body image, sexual functioning, and romantic attachment measures

Measure	Minimum	Maximum	*M*	*SD*
BAS	1	5	3.62	.50
FSFI desire	1.2	6	3.81	.94
FSFI arousal	0	6	4.46	1.76
FSFI lubrication	0	6	4.75	1.97
FSFI orgasm	0	6	4.26	1.87
FSFI satisfaction	.8	6	4.50	1.61
FSFI absence pain	0	6	4.28	2.12
ECR-RS AANP	1	7	2.79	1.54
ECR-RS AAVP	1	7	2.23	.97

*BAS* Body Appreciation Scale, *ECR-RS* Experiences in Close Relationships-Relationship Structures Questionnaire with *AANP* Attachment Anxiety romantic Partner, *AAVP* Attachment Avoidance romantic Partner, *FSFI* Female Sexual Function Index

#### Sexual Functioning

The Dutch version (ter Kuile, Brauer, & Laan, 2006) of the Female Sexual Function Index (FSFI) (Rosen et al., 2000) was used to assess the key dimensions of sexual function in women. It consists of 19 items grouped into six domains: desire (two items), arousal (four items), lubrication (four items), orgasm (three items), satisfaction (three items), and the absence of pain (three items). Each item was scored on a scale of 0 or 1–5. Domain scores were obtained by adding the scores of the individual items that comprise the domain and multiplying the sum by the domain factor (desire .6, arousal and lubrication .3, orgasm, satisfaction, and absence pain .4) (Rosen et al., 2000). Higher scores indicated better and more consistent sexual functioning, while a score of zero indicated no sexual activity during the last 4 weeks. An example of an item is: “Over the past 4 weeks, how often did you feel sexual desire or interest?” The scale was found to be internally consistent with *α* = .82 (Rosen et al., 2000). Dutch research has supported the reliability and psychometric validity of the FSFI and its subscales in adult women (ter Kuile et al., 2006). In the present study, reliability of the total scale was high (*α* = .97). Reliability was also good for all subscales (*α* ≥ .76). Means and *SD*s for the current sample are shown in Table 1.

#### Romantic Attachment

The Experiences in Close Relationships-Relationship Structures Questionnaire (ECR-RS) (Fraley et al., 2011) was used to measure attachment orientation in romantic relationships. Nine items were used, with six items measuring attachment-related avoidance and three items measuring attachment-related anxiety. Responses were measured on a 7-point *strongly* *disagree*-*strongly agree* Likert scale. Mean scores were computed for avoidance and anxiety separately. Higher scores are indicative of higher attachment insecurity. An example of an attachment avoidance related item is: “I don’t feel comfortable opening up to my partner.” An example of an attachment anxiety related item is “I often worry that this person doesn’t really care for me.” Previous studies revealed good reliability for both attachment avoidance (*α* ≥ .81) and attachment anxiety (*α* ≥ .83) (Fraley et al., 2011). The Cronbach’s alphas for avoidance and anxiety in the present sample were .84 and .88, respectively. Means and *SD*s for the current sample are shown in Table 1.

### Statistical Analysis

A structural equation model was fitted to the data using Mplus, version 6.11 (Muthén & Muthén, 2010). The model included nine hypothesized latent variables: attachment avoidance, attachment anxiety, body appreciation, desire, arousal, lubrication, orgasm, satisfaction, and the absence of pain. The structural part of the structural equation model consisted of all the hypothesized relationships between these latent variables. The latent variables desire, arousal, lubrication, orgasm, satisfaction, and the absence of pain were regressed on both attachment variables and on body appreciation. Body appreciation was regressed on attachment anxiety only. The two attachment variables were unexplained by the model (the exogenous variables). The measurement part of the structural equation model consisted of three standard confirmatory factor models. In the first confirmatory factor model, six avoidance items (out of nine) of the ECR-RS only had a factor loading on attachment avoidance, and the three anxiety items only had a factor loading on attachment anxiety. In the second confirmatory factor model, all items of the BAS had a factor loading on the single latent variable body appreciation. In the third confirmatory factor model, the items of the FSFI loaded on the a priori factors. Two items of the FSFI only had a loading on desire, four other items only had a loading on arousal, another four items only had a loading on lubrication, three other items only had a loading on orgasm, another three items only had a loading on satisfaction, and another three items only had a loading on the absence of pain. The structural model and the three standard confirmatory factor models together were fitted to the data as a single structural equation model.^1^

## Results

### Structural Equation Model

**Fig. 1:**
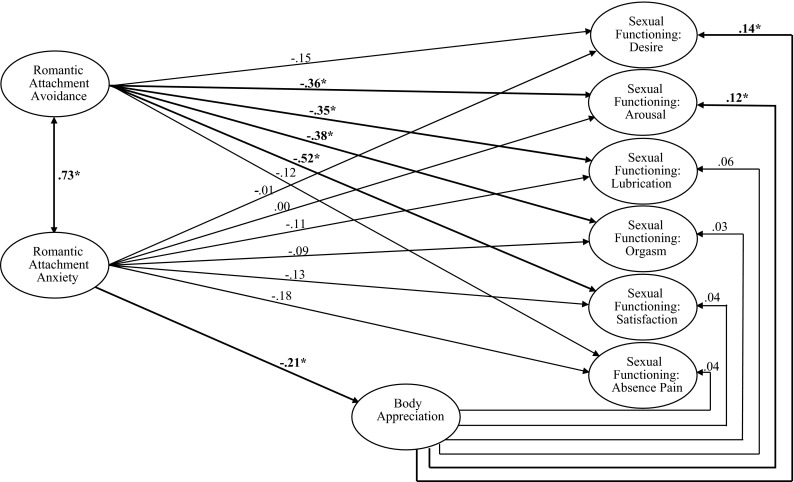
The structural model linking anxious and avoidant romantic attachment with body appreciation and the sexual functioning domains and its parameter estimation results, with *p < .05

Model fit was evaluated using the values of a mean and variance adjusted chi square test statistic, the root mean square error of approximation (RMSEA), Bentler’s comparative fit index (CFI), the Tucker-Lewis Index (TLI), and the Weighted Root Mean Square Residual (WRMR). Since all items were ordered categorical, the structural equation model was fitted to the data using robust weighted least squares estimation. The value of the likelihood ratio chi square goodness of fit statistic was 1378.73 on 744 degrees of freedom (*p* < .001). The estimate of the RMSEA was .047, CFI and TLI were both .98, and WRMR was 1.15. These results indicated a good fit (Schreiber, Nora, Stage, Barlow, & King, 2006). Parameter estimation results are shown in Fig. 1 and in Table 2. Correlations between the sexual functioning domains are not shown in the model, since this was unrelated to the hypotheses of this research. These estimates are shown in Table 2.

**Table 2:** Estimated regression parameters and correlations with standard errors, critical ratios (estimate/standard error), and significance levels between all variables in the structural equation model

Regression of		on/with	Estimate	SE	Est./SE	*p* value
BAS	→	ECR-RS RAAN	−.21	.06	−3.55	<.001*
ECR-RS RAAN	↔	ECR-RS RAAV	.73	.03	25.25	<.001*
FSFI desire	→	BAS	.14	.05	2.57	.010*
	→	ECR-RS RAAN	−.01	.11	−.09	.936
	→	ECR-RS RAAV	−.15	.11	−1.40	.163
	→	FSFI arousal	.57	.05	12.37	<.001*
	→	FSFI lubrication	.45	.05	8.47	<.001*
	→	FSFI orgasm	.32	.06	5.45	<.001*
	→	FSFI satisfaction	.51	.06	9.18	<.001*
	→	FSFI absence pain	.32	.06	5.74	<.001*
FSFI arousal	→	BAS	.12	.06	2.04	.042*
	→	ECR-RS RAAN	.00	.10	.00	.997
	→	ECR-RS RAAV	−.36	.10	3.59	<.001*
	↔	FSFI lubrication	.90	.02	61.29	<.001*
	↔	FSFI orgasm	.59	.04	15.87	<.001*
	↔	FSFI satisfaction	.85	.02	39.93	<.001*
	↔	FSFI absence pain	.65	.04	18.20	<.001*
FSFI lubrication	→	BAS	.06	.06	1.01	.313
	→	ECR-RS RAAN	−.11	.10	−1.10	.273
	→	ECR-RS RAAV	−.35	.10	−3.51	<.001*
	↔	FSFI orgasm	.58	.04	16.27	<.001*
	↔	FSFI satisfaction	.77	.03	26.88	<.001*
	↔	FSFI absence pain	.73	.03	22.80	<.001*
FSFI orgasm	→	BAS	.03	.06	.50	.614
	→	ECR-RS RAAN	−.09	.10	−.88	.379
	→	ECR-RS RAAV	−.38	.10	−3.81	<.001*
	↔	FSFI satisfaction	.51	.04	13.00	<.001*
	↔	FSFI absence pain	.37	.05	7.72	<.001*
FSFI satisfaction	→	BAS	.04	.06	.64	.525
	→	ECR-RS RAAN	−.13	.10	−1.31	.190
	→	ECR-RS RAAV	−.52	.11	−4.86	<.001*
	↔	FSFI absence pain	.62	.04	15.11	<.001*
FSFI absence pain	→	BAS	.04	.06	.63	.530
	→	ECR-RS RAAN	−.18	.11	−1.69	.092
	→	ECR-RS RAAV	−.12	.10	−.18	.237

Single arrows represent one-way paths and double arrows represent correlations

BAS = Body Appreciation Scale; FSFI = Female Sexual Function Index; ECR-RS = Experiences in Close Relationships-Relationship Structures Questionnaire with RAAN = Romantic Attachment Anxiety and RAAV = Romantic Attachment Avoidance

With reference to the hypotheses, the findings were as follows. Hypothesis 1 was partly supported. Body appreciation predicted higher sexual desire and arousal, but none of the other domains of sexual functioning. Consistent with Hypothesis 2, romantic attachment anxiety predicted lower body appreciation in the model. Hypothesis 3 was not supported, since the sexual functioning domains were not predicted by romantic attachment anxiety. Romantic attachment avoidance was directly predictive of lower sexual arousal, less lubrication, orgasm difficulties, and less sexual satisfaction, as expected in Hypothesis 4. However, the desire and absence of pain domain of sexual functioning were not predicted by attachment-related avoidance. Hypothesis 5 was supported, since romantic attachment anxiety and avoidance were associated in the model.

The model indicated significant associations between body appreciation, the romantic attachment dimensions, and the sexual functioning domains. In the model, the body appreciation and the romantic attachment variables explained 4.9 % of the variance in sexual desire scores, 13.5 % of the variance in arousal scores, 16.7 % of the variance in lubrication scores, 16.8 % of the variance in orgasm scores, 28.5 % of the variance in satisfaction scores, and 7.4 % of the variance in absence of pain scores.

## Discussion

The goal of this study was to shed light on links of romantic attachment (i.e., attachment avoidance and anxiety) with positive body image and sexual functioning. We tested a model specifying the relations between romantic attachment avoidance and anxiety, body appreciation, and sexual functioning in young adult females and found a good fit, demonstrating the importance of romantic attachment for both constructs.

Our model confirmed the expectation of relationships between positive body image, sexual functioning, and romantic attachment orientations in young female university students. First, results revealed that body appreciation was associated with attachment related anxiety in relation to a romantic partner, with lower levels of attachment anxiety relating to greater levels of body appreciation. This is in line with findings from another study in which general adult attachment was linked to body appreciation (Iannantuono & Tylka, 2012). Body appreciation, in turn, was associated with sexual functioning, as it was related to higher levels of sexual desire and subjective arousal. Findings further demonstrated a direct relationship between romantic attachment avoidance and sexual functioning. Specifically, lower levels of attachment avoidance were associated with more subjective sexual arousal and vaginal lubrication, higher ability to reach orgasm, and sexual satisfaction.

Taken together, these results suggest both direct and indirect links between romantic attachment and sexual functioning. Whereas low attachment avoidance is likely to lead to a better sexual response and more satisfaction with sexual activity with a partner, low attachment anxiety is likely to enhance positive body image, which, in turn, facilitates better sexual functioning by improving sexual desire and arousal.

It is noteworthy that the attachment and body appreciation variables differed significantly in how much of the variance of the sexual functioning domains they predicted (i.e., 4.9 % of desire, 13.5 % of arousal, 16.7 % of lubrication, 16.8 % of orgasm, 28.5 % of satisfaction, and 7.4 % of absence of pain). Previous research indicated that lack of emotional well-being and negative emotional feelings during sexual interaction with one’s partner are more important determinants of sexual distress (i.e., distress or worry with respect to one’s own sex life) than impairment of the more physiological aspects of female sexual response (Bancroft, Loftus, & Long, 2003). The absence of sexual distress is closely related to sexual satisfaction (Stephenson & Meston, 2010b). For many women, the level of sexual satisfaction is not only based on genital responses during sexual activity with a partner, but also on trust, intimacy, respect, communication, affection, and pleasure from sensual touching (Basson, 2000). Body-related and attachment related feelings may therefore be most strongly linked to affective and emotional components of sexual satisfaction. The weak relationship of body appreciation and attachment variables with sexual desire may be explained by the measurement of sexual desire, the FSFI desire subscale, we used in the current study. Sexual desire can be experienced “spontaneously” in the form of sexual thoughts, sexual dreams, and fantasies, or in response to sexual cues. For many women, sexual arousal and a responsive-type of desire occur simultaneously at the start of sexual activity with a partner (Basson, 2000). Sexual thoughts, dreams, and fantasies, which is what is predominantly referred to in the participant instruction of the FSFI, are experiences that are likely to be less related to factors susceptible to interpersonal influences such as body image and attachment.

Overall, the findings of our model were generally consistent with previously examined links between body image, sexual functioning, and adult attachment (e.g., Cash et al., 2004; Davis et al., 2006). However, our results did not confirm findings of Birnbaum (2007) since attachment anxiety was not directly associated with areas of sexual functioning in the model. Furthermore, our results did not fully confirm the findings of Satinsky et al. (2012), who found that body appreciation positively predicted the arousal, orgasm, and satisfaction dimensions of sexual functioning. This may be explained by differences in sample characteristics like differences in age and sexual orientation or cultural differences between Northern European countries such as the Netherlands and other Western countries (van den Brink et al., 2013). Lastly, the association between romantic attachment anxiety and romantic attachment avoidance was relatively high in our sample, whereas these dimensions were found to be only weakly related in other studies (e.g., Davis et al., 2006).

As discussed by Fraley et al. (2011), it is often assumed by researchers that the anxiety and avoidance dimension should be unrelated based on theoretical considerations. This assumption might be too strong, since the dimensions are separable and conceptually independent. The fact that anxiety and avoidance are relatively highly interrelated does not imply considerable overlap between the anxiety and avoidance dimension (Fraley et al., 2011), but is likely that women who fear intimacy in a specific relationship also tend to avoid closeness and dependency in this relationship. In spite of that, it is possible that anxiety and avoidance often go together in actual practice, the high correlation between the dimensions in our sample may also be explained by measurement choice and sample characteristics. Results of a recent meta-analysis showed that the anxiety-avoidance association was higher among samples using the ECR-R compared to the former version (ECR; Brennan et al., 1998), in samples collected outside of North America, and in samples with more participants in committed relationships (Cameron, Finnegan, & Morry, 2012).

There were several limitations to this study that future research could address. Our sample consisted exclusively of female university students and women with any sexual experience, and was somewhat overrepresented by non-religious participants and underrepresented by overweight participants. Previous research also suggests that women who volunteer to participate in sex research tend to be more sexually experienced, hold less traditional sexual attitudes, and report higher sexual self-esteem (Wiederman, 1999). Therefore, the results of this study may not be representative for the general Dutch population of young women. Additionally, all the variables were measured by self-report, so there could be a self-report bias, most importantly with respect to self-reported weight. Larson, Ouwens, Engels, Eisinga, and Van Strien (2008) found, in a Dutch sample, that heavier female college students tended to underestimate their weight strongly, which can lead to erroneous prevalence estimates of overweight. It is possible that the same trends in relation to inaccurate reporting of weight would apply to the women in our study. Furthermore, other variables that may impact relationships between positive body image, sexual functioning, and romantic attachment orientations were not included in this study. The presence of depressive symptoms among young women, for example, is associated with body dissatisfaction (Stice et al., 2000), problems when engaging in sexual activity with a partner (Frohlich & Meston, 2002), and adult attachment insecurity (Wei, Mallinckrodt, Larson, & Zakalik, 2005). Furthermore, a limitation of our single-item measure of having a romantic partner is that it did not fully validate the relationship as a committed relationship. Committed romantic relationships and dating behavior are both prevalent in college students (Siebenbruner, 2013). Therefore, it is possible that participants who reported having a romantic partner were not (yet) in a committed relationship, reflecting exclusivity, trust, and commitment that can enhance emotional closeness and attachment (Banker, Kaestle, & Allen, 2010). Since duration of partnership and relationship quality were found to be related to romantic attachment (Davis et al., 2006; Feeney, 2004), body image (Ambwani & Strauss, 2007; Weller & Dziegielewski, 2005), and aspects of sexual functioning (Davis et al., 2006; Klusmann, 2002; Murray & Milhausen, 2012), it would be valuable to include these variables in further research. Lastly, given the correlational nature of this study, direction of causation could not be determined. It is also possible that the direction of the relationships is reversed. Women who experience poor sexual functioning may develop less body appreciation and more attachment-related concerns towards their partners (e.g., fear of rejection or discomfort with depending on their partners).

Overall, the findings indicated that attachment security is meaningfully linked to sexual functioning in female university students in two ways. Low levels of attachment anxiety were favorable for experiencing more body appreciation and, in turn, more sexual desire and subjective sexual arousal. Low levels of attachment avoidance were directly linked with better sexual functioning, by enhancing more arousal and vaginal lubrication, higher ability to reach orgasm, and sexual satisfaction. To our knowledge, this was the first study to examine the role of romantic attachment in relationships between positive body image and sexual functioning. As highlighted by Satinsky et al. (2012), the discovery that positive body image is related to sexual function offers implications for promoting sexual health moving away from the traditional focus on negative body image. The finding that romantic attachment is meaningfully linked with positive body image and sexual functioning can help make a step-change in understanding and treating mental health issues in the context of sexual dysfunction.

The development of an attachment relationship towards a romantic partner is an important developmental task during early adulthood marking the transformation of dating to committed romantic relationships (Arnett, 2000). Since adult attachment is strongly based on attachment experiences earlier in life (e.g., Bowlby, 1969), it might be difficult for young women who were insecurely attached as children and adolescents to use their romantic partners as a “secure base.” These young women may therefore be more likely to engage in (casual) sexual relationships without commitment, with potential risk for experiencing negative consequences, such as sexual assault (Littleton, Tabernik, Canales, & Backstrom, 2009). As many university health services increase their focus on mental health issues, the role of romantic attachment can be a continued area of interest for researchers and clinicians. In clinical settings, we recommend to pay attention to the intimate relational context through, for example, involving romantic partners in individual treatment programs. Furthermore, we feel that emotionally focused couple therapy is useful in this respect. This form of therapy can be effective in targeting negative interactions between partners that maintain attachment insecurity, reprocessing negative emotional experiences of sex, and develop a more secure bond with the partner in order to build on a more satisfying sex life (Johnson & Zuccarini, 2010). The associations between body appreciation, sexual functioning and romantic attachment underline that specific (elements in) treatment programs can be helpful in building on a positive cycle, in which positive body image, a satisfying sex life, and a secure bond with the partner can reinforce each other.

## Footnotes

1 As pointed out by Meyer-Bahlburg and Dolezal (2007), there are issues with administering the FSFI in samples with lower rates of sexual activities. Therefore, we assessed whether there were differences between respondents who reported being sexually active with a romantic partner and respondents not currently engaged in a romantic relationship. Multi-group versions of the structural equation model under the requirement of measurement invariance were fitted to the data. Chi square difference test results revealed that there were no significant differences in the structural relationships among the latent variables between the two groups. Therefore, the total sample was used in the structural equation model.
